# Multiple signaling pathways in the frontiers of lung cancer progression

**DOI:** 10.3389/fimmu.2025.1593793

**Published:** 2025-06-10

**Authors:** Hao Liu, Dujuan Zhou, Yangjing Ou, Shuanghua Chen, Yunzhu Long, Ting Yuan, Yukun Li, Yingzheng Tan

**Affiliations:** ^1^ Zhuzhou Clinical College, Jishou University, Zhuzhou, Hunan, China; ^2^ Department of Infectious Disease, Zhuzhou Hospital Affiliated to Xiangya School of Medicine, Central South University, Zhuzhou, Hunan, China; ^3^ Hunan Traditional Chinese Medicine College, Zhuzhou, Hunan, China; ^4^ Department of Assisted Reproductive Centre, Zhuzhou Hospital Affiliated to Xiangya School of Medicine, Central South University, Zhuzhou, Hunan, China

**Keywords:** signal transduction, lung cancer, therapeutic targets, oncogene, metastasis

## Abstract

Lung cancer is a prevalent malignant tumor and the leading cause of cancer-related mortality worldwide. LC is a complex respiratory condition that poses significant challenges for both clinicians and researchers. Crucially, dysregulation of molecular signaling pathways is a key message point in LC. Numerous reviews have highlighted effective treatments for LC by targeting disrupted signaling pathways. Understanding the roles and interconnections of various signaling pathways in LC is crucial. Therefore, this paper reviews the pathogenesis, biological functions and their important interactions in lung cancer. Frist, we reviewed relevant signaling pathways involved in LC, including Wnt, PI3K/Akt, Notch, PD-1/PD-L1, NF-κB, Hippo, MAPK, Hedgehog, AMPK. Immediately thereafter, we further explored the biological functions of LC in this area of pathophysiology, such as apoptosis, metastasis and proliferation. In conclusion, after our deeper understanding of the interactions of these signaling pathways in LC. And we must recognize that the interactions between the above signaling pathways can lead to comprehensive as well as novel therapeutic approaches for LC.

## Introduction

Lung cancer (LC) is one of the most prevalent malignant tumors in the world, noted for its high incidence and mortality rates (1, 2). The main reasons for this outcome are the absence of early symptoms of LC and the insufficient emphasis on cancer prevention strategies in developing countries, leading to unexpected global harm (1, 3). Although surgical resection has some clinical efficacy in early-stage LC, treatment options and survival are limited by late symptom onset, restricted screening platforms, and limitations of conventional radiotherapy and chemotherapy for advanced LC (4).

LC is a complex respiratory condition that poses significant challenges for both clinicians and researchers (5). Various studies have shown that Inflammation, dysbiosis of lung and gut microbiota, genetic mutations, metabolic disorders, immune dysfunction, epigenetic modifications, oxidative stress, genetic factors and aberrant hormone expression are major risk factors for LC (6–13). During host defense or therapeutic injury, various molecular mechanisms are modified, such as mitophagy (14), mucin expression anomalies (15), epithelial–mesenchymal transition (EMT) (16), reactive oxygen species (ROS) generation (17), angiogenesis (18), abnormal glycosylation (19), and processes like cell apoptosis, proliferation, survival, metastasis, and invasion. The dysregulation of molecular mechanisms alone appears insufficient to fully explain the origins and progression of LC, and it is reasonable to suspect that genetic and epigenetic events have had an impact on genetic aspects (20, 21).

The functions and interactions of molecular pathways are involved in a wide range of cancers and have significant effects. Research indicates that the dysregulation of various signaling pathways, including Wnt (22), PI3K/Akt (23, 24), Notch (25), PD-1/PD-L1 (4), NF-κB (26), Hippo (27), MAPK (17, 28), Hedgehog (Hh) (29), AMPK (30), can facilitate LC progression and metastasis. Furthermore, the interactions between these pathways are precise and complex. Numerous studies indicate that genetic and epigenetic disturbances both contribute to and result from the development of LC (31, 32). Cristian et al. found that arsenic ingestion can cause cancer by producing epigenetic modifications and disrupting normal microRNAs (miRNAs) expression (33). Gao et al. the study indicated that radicicchioidin (SFN) could potentially prevent LC by restoring miR-9–3 levels through the inhibition of DNMTs, HDACs, and the target gene CDH1 protein levels (34). Yang and colleagues elucidate the role of epigenetic generation in LC progression (35).

In the current review, we provide a deep understanding and summary of the formation of the molecular mechanisms of these studies and the impact they have on LC, which we believe will provide a more effective and comprehensive therapeutic strategy for LC.

## Multiple oncogenic and anticancer intracellular pathways in LC

We summarize recent advances in LC in the desire to gain a deep understanding of the molecular pathogenesis of LC (**Table 1**). Research has shown that intracellular signaling pathways can induce oncogenic effects, and targeting the driver genes within these pathways is highly effective in tumor treatment. Furthermore, expanding research on molecular diseases in LC offers crucial insights into its carcinogenesis, elucidated by various molecular mechanisms that function diversely across cancer development stages or contexts.

**Table 1:** The roles of the following signaling pathways in LC and associated inhibitors and activators.

Signaling pathways	Function	Inhibitors	Activators
Wnt PI3K/Akt	Promoting metastasis, proliferation Inhibition apoptosis (36, 37) Regulation DC, T cell, B cell, NK cell and macrophage functions (38) Promoting proliferation,metastasis, survival and angiogenesis (25, 39) Regulation macrophage functions (40)	Atranorin Thioridazine hydrochloride	BML-284 ELA-32
Notch	Promoting proliferation, metastasis, angiogenesis and EMT (41–43)	DAPT	Sodium Valproate
	Regulation T cell and B cell functions (44, 45)		
PD-1/PD-L1	Promoting immune evasion, metastasis (46) Regulation T cell functions (46)	Camrelizumab	D18
NF-kB	Promoting proliferation, survival, metastasis and angiogenesis and regulating inflammatory response (47–49) Regulation DC, T cell, B cell and macrophage functions (48, 50, 51)	Andrographolide	TNF-α
Hippo	Inhibition metastasis, proliferation, invasion and EMT and regulating organ size (52, 53) Regulation macrophage functions (54)	Verteporfin	PY-60
MAPK	Promoting self-renewal, metastasis (55, 56) Regulation T cell and B cell functions (57, 58)	Curcumin	N-Methylparoxetine
Hedgehog	Promoting proliferation, angiogenesis, metastasis (59, 60) Regulation T cell and macrophage functions (61)	Cyclopamine	SAG
AMPK	Promoting apoptosis and Inhibition proliferation, metastasis and Regulating energy matabolism (62–64) Regulation DC, T cell, B cell, neutrophil and macrophage functions (65)	Dorsomorphin	AICAR

### Wnt pathway

The Wnt pathway consists of two canonical and noncanonicall types, one of which is the classical pathway in which Wnt binds to the LRP-5/6 receptor (LDL receptor) and Frizzled receptor, resulting in Disheveled (DVL) being phosphorylated, allowing the complex (Axin, GSK-3β, CK1, APC) to inhibit the activity of GSK-3β and reduce β-catenin ubiquitination and proteasomal degradation, allowing enrichment of unphosphorylated β-catenin (66–68). Unphosphorylated β-catenin translocated to the nucleus binds to TCF/LEF and induces the expression of multiple target genes (66–68).

When Wnt binds to Frizzled receptors and interacts with Daam2, it initiates a Wnt non-canonical pathway called planar cell polarity (PCP). This interaction activates small GTPases like RhoA and Rac, subsequently triggering downstream stress kinases JNK and ROCK, which play roles in cytoskeletal remodeling and actin alignment (69). Wnt/Ca2+ pathway is another Wnt noncanonicall pathway that is activated by Wnt5a and Fzd2 receptors, leading to G protein-mediated activation of PLC, which allows for a large amount of Ca2+ inward flow. The increased intracellular Ca2+ then stimulates calmodulin phosphatase and CAMKII, which in turn promotes TCF phosphorylation, which inhibits the Wnt classical pathway (70, 71).

The Wnt signaling pathway is a key regulator of LC development, metastasis, and drug resistance, as identified in previous studies (36, 37, 72). The study identified that isoform 1 of the neurogenesis-associated protein ASPM (ASPM-I1) plays a crucial role in SCLC development by stabilizing the Hh transcription factor GLI3 via a unique coding region in exon 3 and activating the Wnt-DVL3-β-catenin signaling pathway, which sustains the transcription of the Hh pathway regulator Smoothened (SMO) (73). Cisplatin (CP) is a widely used chemotherapeutic agent, and studies have shown that miRNAs can trigger the Wnt/β-catenin signaling pathway, leading to varying degrees of resistance to this chemotherapeutic agent in LC cells (74). In addition, it has been found that a variety of non-coding RNAs (ncRNAs) can regulate the expression of the Wnt/β-catenin signaling pathway, which affects the progression of LC to varying degrees, and thus targeting ncRNAs appears to be a good gene therapy (75). Studies have shown that Wnt can be mutually promoted with PD-L1, suggesting that Wnt can be controlled by PD-L1 (76), PD-L1 is important for lung tumorigenesis and progression in mice by regulating Yes-associated protein (YAP) (77). And it is the potential mechanism of the Wnt pathway on the PD-1/PD-L1 pathway that makes non-small cell lung cancer (NSCLC) patients somewhat resistant to immune checkpoint inhibitors (ICIs), so it seems that the combination of Wnt inhibitors and ICIs could be a new option for the treatment of LC patients (78). The study revealed that fibronectin overexpression in LC activates FAK and MAPK/ERK signaling pathways, which in turn overactivate the Wnt pathway, facilitating tumor progression (79).

Tumor metastasis is a leading factor in poor prognosis for LC patients (80), with the Wnt signaling pathway significantly contributing to its development (36). In addition, metastasis of cancer cells is not a one-sided problem, but requires the acquisition of a series of conditions to realize this process (81). Among other things, we found that tumor cells are mainly powered by uptake of stromal cells and immune cells from the tumor environment during this process (82, 83). Research indicates that the tumor microenvironment (TME) significantly influences tumor progression, with various specialized microenvironments within the TME interacting with cancer (84). Notably, hypoxia is a critical factor in tumor development (85). Research indicates a link between cancer incidence and chronic inflammation, with microenvironmental dysregulation causing persistent inflammatory lesions, reinforcing the validation that TME after tumor metastasis contributes to LC development (80, 86, 87). While Wnt signaling seems to interact with TME by regulating different components of TME (88). In addition, the Wnt signaling pathway can be involved in the functioning of a variety of immune cells such as dendritic cells (DCs), T cells, B cells, natural killer (NK) cells, macrophages, granulocytes, etc, which is the main cause of cancer, but it also provides us with a good strategy for immunotherapy (38). Cancer stem cells (CSCs) can promote lung tumor metastasis, and studies have found that by targeting the Wnt pathway associated with CSCs, it seems to have good efficacy in the treatment of LC (29, 89). Latency competent cancer (LCC) cells are cells that can enter the quiescent state very easily, and we found that the main mechanism is that the Wnt signaling pathway leads to the ability of LCC cells to escape from NK cell immunosurveillance through autocrine DKK1 (90). In addition, overexpression of Metadherin (MTDH) upregulates the Wnt pathway and depletes cytotoxic T cells, which promotes LC metastasis and progression (91, 92).

### PI3K/Akt pathway

Inhibiting the PI3K/AKT signaling pathway, crucial for LC progression, could offer a significant therapeutic strategy (93). PI3K is a lipid kinase that converts phosphatidylinositol-4,5-bisphosphate (PIP2) into the second messenger phosphatidylinositol-3,4,5-trisphosphate (PIP3), which facilitates Akt translocation to the endosomal membrane for phosphorylation and thus facilitates signal expression (94). AKT functions downstream of PI3K, with PIP3 signaling being terminated by the lipid phosphatase PTEN. The PI3K/AKT pathway, often dysregulated in human cancers, contributes to cancer cell growth and metastasis (95).

This pathway phosphorylates NF-κB to enhance cell survival by having IKK phosphorylate inhibitory IκBα, facilitating NF-κB’s nuclear translocation to promote cell survival and vascular production, thereby inducing oncogenesis (39, 96). This pathway enhances angiogenesis in LC by modulating vascular endothelial factor (VEGF), leading to hypoxia-inducible factors (HIFs)-1α binding to the HRE in the VEGF promoter region (25, 97). Arsenic and benzo[α]pyrene (BaP), key contributors to LC, enhance integrin α4 (ITGA4) expression, thereby activating the PI3K/AKT pathway (98). This activation reduces suppressor of fused (SUFU) protein stability and concentration, allowing the Hh ligand to bind Patched (Ptch) and release SMO. Consequently, the GLI transcription factor is expressed, activating the Hh pathway and significantly increasing CSCs properties and tumorigenesis (98). This pathway regulates the EMT process, one of the major molecular mechanisms regulating lung tumor metastasis, which acts by inhibiting E-calmodulin expression and upregulating mesenchymal markers and EMT-specific transcription factors (99, 100). Long non-coding RNA (lncRNA) significantly regulates the PI3K/AKT signaling pathway in LC (101, 102). Specifically, lncRNA FOXD3-AS1 is highly recruited by exosomes in LC cells, where it interacts with ELAV-like RNA-binding protein 1 (ELAVL1) to activate this pathway, promoting LC progression (103). The study identified that Go-Ichi-Ni-San complex subuint 2 (GINS2) enhances phosphorylated proteins via PI3K/AKT and MEK/ERK pathways, thereby facilitating NSCLC growth, metastasis and EMT in mice (104). Wang et al. identified that CXCL5, a chemokine associated with NSCLC prognosis, is overexpressed in LC cells and to enhance LC progression through the activation of PI3K/AKT and MAPK/ERK1/2 signaling pathways (105). Radiation therapy is one of the main options for the treatment of various types of cancer, and it destroys cancer cells by means of ionizing radiation (IR) (106). However, we found that while destroying cancer cells, IR induces ROS generation and EMT, and to a certain extent leads to changes in TME and thus promotes metastasis, and it has been found that targeting the PI3K/Akt pathway seems to enhance the efficacy of the treatment by inhibiting EMT (107). KRAS G12D mutation in LC was found to activate the PI3K/Akt pathway and thus promote LC progression (108). In contrast, Hou et al. found that Salvianolic acid F (SalF) inhibits PI3K/Akt pathway expression by targeting KRAS G12D mutants (109). In addition, in NSCLC patients with PI3K/Akt pathway activation combined with epidermal growth factor receptor (EGFR) mutations, it was found that the combination of inhibitors of this pathway with EGFR Tyrosine Kinase Inhibitors (TKIs) improved the resistance of such patients to EGFR-TKIs (110). It was found that the Akt pathway can influence metabolic signaling and convergent inflammation to regulate macrophage function and can promote its M1/M2 polarization (40). Research on the PI3K/AKT pathway’s impact on immune cells and TME regulation revealed that combining immunotherapy with targeted therapy enhances efficacy in LC patients (111).

The PI3K/Akt pathway activates mTOR by inhibiting tuberous sclerosis complex 1 (TSC1), enhancing protein synthesis and promoting cellular metabolism, growth, and proliferation, thereby regulating cancer cell growth and metastasis (39). Collectively, the findings indicate that this pathway facilitates LC growth and metastasis.

### Notch pathway

The Notch signaling pathway, comprising four receptors (Notch1-4) and five ligands (Delta-like 1, 3, 4, and Jagged 1, 2), plays a crucial role in regulating the biological functions of LC cells (112).

Various studies have shown that this pathway promotes the progression of LC through multifaceted effects (41, 113). Initially, ADAM10 hydrolyzes Notch protein near the membrane, followed by γ-secretase cleavage of the Notch intracellular domain (NICD) (113). This cleavage allows NICD to translocate into the nucleus, where it binds to CBF-1/suppressor of hairless/Lag1 (CSL), thereby promoting the transcription of downstream targets (41). RFC4 enhances the Notch signaling pathway by binding to NICD1, thereby preventing CDK8/FBXW7-mediated phosphorylation and polyubiquitination (114). Delta-like ligand 3 (DLL3), a Notch inhibitory ligand, is markedly up-regulated on SCLC cell surfaces, enhancing cell growth, metastasis and proliferation, which contributes to resistance against platinum-based chemotherapy (115). Therefore, against the high expression of DLL3, it was found that Tarlatamab (AMG 757) could cause tumors to regress to different degrees by combining DLL3 and CD3 on T cells, which provides a new direction for targeted immunotherapy for SCLC (116). In addition, we found that the chimeric antigen receptor (CAR) for DLL3 has an antitumor effect in SCLC in mice, and thus therapies targeting CAR T cells for DLL3 may provide a new strategy for the treatment of SCLC (117). Notch and Wnt/β-catenin signaling pathways are closely interconnected, as β-catenin enhances Notch signaling by binding to the Dll4 promoter, which subsequently influences the Wnt/β-catenin pathway through Nrarp regulation (118).

Numerous studies indicate that the Notch pathway is upregulated in LC patients, leading to cancer cell proliferation, metastasis, EMT and angiogenesis (41–43). For the Notch pathway to promote metastasis in cancer cells, a key point is that it promotes angiogenesis and appears to be related to the ability of the pathway to induce EMT (119). In cancer cells undergoing distant metastasis, aberrant angiogenesis in TME mainly plays a role in providing energy to cancer cells (120). The promotion of tumor angiogenesis by the Notch pathway is primarily attributed to the roles of ligands DLL4 and JAG1 (121, 122). Furthermore, EMT is a key factor contributing to chemotherapy resistance in NSCLC (123). Lu et al. found that the ADAM17 inhibitor ZLDI-8 could inhibit the Notch pathway and EMT thereby significantly promoting apoptosis in chemotherapy-resistant NSCLC and inhibiting NSCLC invasion and metastasis (124). SCLC has a poor prognosis due to its resistance to chemotherapeutic agents (125). Research on targeted therapies revealed that LSD1 inhibitors can impede SCLC progression by targeting Notch signaling and reducing ASCL1 transcription factor expression (126). The Notch pathway is crucial for T cell differentiation and B cell development (44, 45). It is well known that the current main strategy of immunotherapy is to modulate the immune system in order to enhance its power to destroy cancer cells (127). Instead, the study reports a novel T-cell therapy that uses synthetic Notch (synNotch) receptors to alter tailored behavior in T cells and control their differentiation, and also specifically targets tumors through such T cells (128).

### PD-1/PD-L1 pathway

The PD-1/PD-L1 pathway is crucial in LC development (46, 129), in which PD-1 is expressed in a variety of immune cells such as T cells and dendritic cells (130), while PD-L1 is present in macrophages, epithelial cells, etc (131). PD-L1 binds to and acts on PD-1 to inhibit T cell expression and facilitating immune evasion (46).

PD-L1 expression in LC patients is regulated by various pathways (132–134). NF-kB directly binds to the PD-L1 promoter to enhance its expression and also promotes HIF-1α transcription (132), which subsequently stimulates glycolysis in lung tumors and up-regulates PD-L1 by modulating glycolytic enzymes (135). YAP proteins act as effectors in the Hippo pathway signaling cascade, which is linked to tumor cell metastasis and proliferation (136). EGFR, a transmembrane tyrosine kinase receptor (137) that phosphorylates Hippo kinase, which enhance YAP protein expression and regulate PD-L1 (133). The study identified an association between chemoresistance in lung squamous cell carcinoma (LUSC) and the interaction of the NRF2 and PD-1/PD-L1 pathways (134). In addition, PD-L1 promotes the expression of hexokinase 2 (HK2) and glycolysis in LC cells thereby inhibiting the function of effector T cells in LC (135). Interleukin-1β (IL-1β) promotes tumor growth and metastasis and appears to synergize with the PD-1/PD-L1 pathway to enhance lung tumor development (138).

PD-1 is currently a well-studied immune checkpoint (ICP) and its ligand, PD-L1, is overexpressed in LC, whereas metastasis of cancer cells occurs by promoting evasion of immune surveillance (46, 139). PD-L1, also referred to as B7-H1, has been identified in prior research as a key mechanism for promoting immune evasion in lung tumor cells by inducing apoptosis in activated tumor-reactive T cells (140). It has been previously stated that the poor prognosis of LC patients is due to the relative limitations of radiotherapy in the treatment of advanced LC (4). With immunotherapy research, targeting the PD-1/PD-L1 pathway and combining it with other therapeutic options has resulted in a significant increase in the survival of patients with advanced LC, making immune checkpoint inhibitors (ICIs) the preferred treatment for advanced LC at this time (4, 141, 142). In addition, for patients with advanced NSCLC, it has been shown that the bispecific antibody AK112 works by targeting both PD-1 and VEGF and can be combined with chemotherapeutic agents to achieve a good anti-tumor effect (143, 144). In addition, gene editing therapy mediated by transcription activator-like effector nuclease (TALEN) can inhibit PD-1 expression in CAR-T cells thereby reducing T-cell depletion and thus prolonging anti-tumor activity (145). Hypoxia significantly contributes to metastasis in advanced cancers, with HIFs serving as central mediators in this process (146). HIFs are overexpressed in hypoxic conditions, enhancing PD-L1 levels, which suggests that inhibiting the HIF/PD-L1 pathway could enhance cancer treatment efficacy (147). It was found that targeting High-mobility group box 1 (HMGB1) could remodel TME and enhance its efficacy in combination with anti-PD-1/PD-L1 immunotherapy for anti-cancer purposes (148). Immunotherapy targeting the PD-1/PD-L1 signaling pathway is a therapeutic option for advanced LC due to its sustained anti-tumor immune response (149).

### NF-κB pathway

NF-κB, a stress-regulated transcription factor from the Rel family, is crucial in connecting inflammation with tumor cell survival (47). The NF-κB signaling pathway is pivotal in both innate and adaptive immune responses and is divided into classical and non-classical pathways (48).

The canonical NF-κB pathway can be activated by various agents, including interferon-based stimulators (STING), interleukin 1 (IL-1), tumor necrosis factor (TNF-α) and numerous drugs (150, 151). IKK activation is usually mediated by IKKβ-promoted IκB phosphorylation, and the activated IKK complex consists of two kinase subunits (IKKα and IKKβ) and a regulatory subunit (IKKγ/NF-κB essential modulator (NEMO)). The IKK complex facilitates IκBα phosphorylation and degradation, enabling NF-κB translocation to the nucleus and induce gene expression (151, 152). The noncanonical NF-κB pathway is primarily mediated by CD40, LTβR and NF-κB receptor activators, which stabilize NF-κB-induced kinase (NIK). This stabilization promotes IκB kinase-α (IKKα) activation, leading to the phosphorylation, ubiquitination, and processing of p100. Consequently, the p52/ReIB NF-κB complex translocate to the nucleus and thus induces gene expression (153).

Furthermore, it was found that STING and NF-κB can promote each other, where the classical NF-κB pathway increases STING expression by regulating microtubule depolymerization (150). NF-κB signaling enhances EMT by upregulating ZEB1/2, transforming growth factor-β and Slug gene transcription, which suppresses the epithelial marker E-calreticulin and promotes N-calreticulin and poikilodulin expression (154). Interleukin-6 (IL-6) is a key cytokine in immune regulation, but its abnormal expression is linked to inflammation and cancer progression (155). Aberrant expression of T-cell immunoglobulin domain and mucin domain 4 (TIM-4) correlates with poor LC prognosis, and IL-6 can upregulate TIM-4 via NF-κB activation, promoting EMT expression and LC development (156). In addition, Xiang et al. found that semaphorin 4A (Sema4A) could promote phosphorylated NF-κB pathway-related proteins as well as IL-6 expression to promote LC cell migration and proliferation (157). Mesoderm-specific transcripts (MEST) were found to induce STAT3 expression to enhance IκBα and P65 phosphorylation activity, and it also promotes IκBα degradation and thus modulates NF-κB signaling by interacting with valine-containing protein (VCP) (158). STAT3 signaling enhances the NF-κB pathway by upregulating miRNAs through interaction with IL-6. These miRNAs regulate various pathways and promote angiogenesis in the NF-κB pathway by inhibiting the deubiquitinating enzyme CYLD (159). In patients with NSCLC, studies have shown that the NF-κB pathway induces PD-L1 expression leading to immune evasion of cancer cells, and thus the combination of NF-κB inhibitors with ICIs appears to be useful for the treatment of NSCLC (160). S-adenosylmethionine (SAM) is a natural metabolite, and recent studies have found that SAM can target P62 and thus inhibit the NF-κB pathway in NSCLC, thus SAM may become a relatively safe adjuvant therapeutic agent (161).

In LC, NF-κB is also one of the important pathways that promote metastasis in cancer cells, in which the induction of EMT expression by this pathway is an important mechanism to achieve this process (154). Among them, the classical NF-κB pathway regulates multiple pro-inflammatory and pro-angiogenic factors, leading to chronic inflammation and promoting angiogenesis (48, 49). Because of this, anti-inflammatory drugs can be used as clinical therapeutic options to inhibit the NF-κB pathway (48). DCs facilitating T cell activation by presenting antigens, thus bridging innate and adaptive immunity (162). Whereas the non-classical NF-κB pathway regulates DC, T and B cell development and plays a role in mediating lymphoid organ development and osteoclast differentiation (48, 50). The infiltration of immunosuppressive macrophages, crucial for primary tumor metastasis, is driven by tumor-derived exosomes (TDE) that polarize macrophages into an immunosuppressive phenotype via NF-κB activation and glycolytic metabolic reprogramming (51). Platelets create a protective barrier for tumor cells against immune cell attacks and boost their metastatic potential by inducing EMT through transforming growth factor-β (TGFβ) (26). Activation of the NF-κB pathway during interactions between platelets and cancer cells can increase pro-metastatic potential (26). In conclusion, the NF-κB signaling pathway’s regulation of immune function facilitates cancer cell metastasis, yet it also offers a crucial target for clinical treatment of LC.

### Hippo pathway

Hippo signaling is one of the important signaling during tumorigenesis and cancer cell development (163), which was first discovered in Drosophila melanogaster and plays an important role in regulating stem cell proliferation, differentiation, migration, apoptosis, organ size and self-renewal (52). In addition, Hippo signaling mainly consists of MST1/2, SAV1, MOB1A/B, LATS1/2, YAP, transcriptional coactivator with PDZ-binding motif (TAZ) and transcription enhancement-associated structural domain (TEAD) family of multiple key proteins (164), and importantly the pathway can inhibit tumor development through these components (163). Activation of the Hippo kinase cascade enhances MST phosphorylation of LATS, which subsequently binds to MOB1 to phosphorylate YAP (165–167). This process sequesters YAP in the cytoplasm, inhibiting the YAP/TAZ complex’s interaction with TEAD (165–167), ultimately contributing to lung tumorigenesis (53).

In LC, dysregulation of the Hippo signaling pathway induces migration, invasion, proliferation, drug resistance and EMT in LC cells (53). It was found that by targeting PFKFB3 (6-phosphofructose-2-kinase) it could down-regulate YAP/TAZ expression to inhibit the glycolytic process of CSCs in SCLC and enhance the chemosensitivity of SCLS (168). In addition, the herbal medicine cryptotanshinone (CT) can regulate TAZ expression to activate Hippo, thereby inhibiting the progression of NSCLC and reducing its chemoresistance (169). YAP upregulates EGFR ligands like Amphiregulin (ARGE) and Neuregulin 1 (NRG-1), creating a positive feedback loop in the MAPK signaling pathway (133). This process also inactivates the oncogene k-ras, which subsequently interacts with FOS to activate MAPK signaling, thereby promoting EMT expression (133, 170). In addition, YAP/TAZ can enhance PD-L1 protein expression to promote NSCLS immune evasion (170).

YAP/TAZ is a major effector downstream of the Hippo signaling pathway that promotes metastasis by reprogramming cancer cells, but the Hippo cascade reaction can ultimately phosphorylate and inhibit YAP/TAZ (136). In addition, we found that YAP/TAZ promoted glycolysis and glutamine catabolism, which provided energy support to metastatic tumor cells (171). YAP has been reported to interact with the transcription factors TEAD and PRDM4 to induce leukocyte-specific integrin β2 (ITGB2) expression thereby mimicking the behavior of leukocyte endothelial invasion and ultimately promoting cancer cell invasion and distant metastasis (172). Mitotic Spindle Positioning (MISP) suppresses MST1/2 activity, resulting in YAP hyperactivation and increased SCL7A11 expression. This process enhances lung cancer cell resistance to ferroptosis, promoting tumor metastasis, yet also offers a potential therapeutic target for LC (173). It is well known that the level of tumor immunogenicity affects the survival of tumor cells, and it has been found that inhibition of LATS1/2 kinase in the Hippo pathway seems to enhance tumor immunogenicity and thus can achieve the purpose of LC treatment (174). The Hippo pathway influences specific immune cells, impacting immunotherapy effectiveness (164). Hippo pathway dysregulation leads to increased YAP activity, which elevates CCL2 and CXCL5 cytokine levels, facilitating the recruitment of M2 macrophages and polymorphonuclear myeloid-derived suppressor cells (MDSCs), thereby contributing to immunotherapy resistance (54, 175).

### Ras/Raf/MEK/MAPK/ERK pathway

The MAPK pathway is crucial in human tumors, influencing cell proliferation, differentiation, apoptosis and angiogenesis. The RAS gene is tumorigenically mutated in approximately 30% of tumors. Activated Ras mediates the activation and phosphorylation of Raf membrane translocation, which in turn promotes MEK activation, which then phosphorylates and activates MAPK/extracellular signaling-associated kinase (ERK) by phosphorylating Tyr and Thr residues (176).

In LC, gene mutations in the MAPK pathway are activated. In EGFR-mutated NSCLC cells, the MAPK pathway enhances PD-L1 expression by modulating drug resistance mechanisms like c-MET amplification and EGFR-T790M mutation, resulting in resistance to EGFR-TKIs (177, 178). Mutations in T790M and C797 within the ATP receptor cause NSCLC to be resistant to EGFR-TKIs, and it has been found that the EAI045 inhibitor works by targeting such mutants and has good efficacy in combination with cetuximab for the treatment of EGFR-TKIs resistant mutants in LC (179). In addition, studies have shown that the inhibitor Sotorasib directly targets the KRAS G12C mutant protein, thereby improving survival in patients with KRAS G12C mutations in NSCLC (180). Fentanyl has been found to reduce the sensitivity of cisplatin (DDP) chemotherapy by inducing a MAPK signaling cascade response through the promotion of ROS expression (181). In addition, the oncoprotein hepatitis B X-interacting protein (HBXIP) was found to be upregulated in NSCLC, which reduces MEK1 protein degradation to promote MAPK/ERK pathway expression (182). In addition, the MAPK/ERK and PI3K/AKT signaling pathways seem to play a synergistic role in promoting self-renewal of lung CSCs during LC development (55). This suggests that the MAPK pathway can interact with the PI3K/AKT pathway in LC.

The MAPK signaling pathway is a central pathway in the regulation of LC metastasis (56). Among the major molecular mechanisms driving LC metastasis is the Ras gene mutation that ultimately triggers the MEK/ERK cascade reaction, leading to extracellular matrix remodeling (ECM) and EMT (183). In addition, the MAPK pathway can enable the formation of a metastasis-promoting immunosuppressive environment by regulating the TME (58, 184, 185). First, this pathway promotes VEGF expression, leading to increased angiogenesis (185). Secondly, mutant KRAS activates the MEK/ERK/AP-1 pathway and thus promotes the expression of TGF-β1 and IL-10. This process recruits regulatory T cells (Tregs), further diminishing the antitumor immune response (58). Moreover, KRAS mutations can also upregulate PD-L1 via the ERK pathway, leading to depletion of T cells, resulting in immune evasion (184). Currently, because KRAS-mutant lung adenocarcinoma (LUAD) is resistant to the MEK inhibitor trametinib, it has been found that trametinib in combination with ICIs for the treatment of LUAD exerts a synergistic anti-tumor effect (186). In addition, early B cell development as well as late B cell maturation are also regulated by the MAPK pathway (57). Despite the current efficacy of ICI in advanced LC (4), it is limited by the fact that patients with KRAS mutations result in immunosuppressive TME formation (187). Therefore, combination therapy is particularly important, and it has been found that KRAS G12C inhibitors combined with ICI can not only inhibit the proliferation of lung tumors but also lift TME immunosuppression (188). In KRAS-mutated lung cancer, combining MEK with CDK4/6 inhibitors suppressed cell proliferation and enhanced NK cell-mediated immunosurveillance (189).

### Hedgehog pathway

Initially discovered in Drosophila, the Hh signaling pathway is crucial for embryonic development regulation (190). The HH protein family is important in the regulation of cell proliferation, apoptosis, differentiation, metastasis and invasion (59). HH proteins (SHH, IHH, or DHH) bind to the PTCH1 receptor, prompting its lysosomal degradation and reducing its repression of Smo (191). This process activates GLI protein expression, causing GLI1 and GLI2 transcription factors to the nucleus and promotes gene expression (192).

Research indicates that the Hh signaling pathway is important in regulating lung tumor cell proliferation, drug resistance, stemness and the tumor microenvironment (193, 194). ASPM-I1 is a stemness gene that is significantly upregulated in SCLC cells, which regulates the activity of the transcription factor GLI1 and promotes SMO transcription through signaling with Wnt-DVL3-β-catenin (73). In addition, it was found that tretinoin could mediate HNF1A/SHH expression to inhibit Hh signaling thereby enhancing the sensitivity of LC cells to paclitaxel (195). In addition, Wang et al. found that SFN inhibited the expression of SHH, SMO and GLI1 in LC cells and thus inhibited LC cell proliferation (196). Research indicates that Sonic Hedgehog (Shh) is upregulated in A549 and H520 cells, enhancing NSCLC angiogenesis by modulating collagen production in fibroblasts (60).

The HH signaling pathway is crucial for promoting metastasis in LC by inducing EMT expression via the metastasis factor Gli, which is the primary mechanism driving cancer cell metastasis (197). Bone is frequently affected by LC metastasis. The HH pathway enhances receptor activator of NFkB ligand (RANKL) expression in osteoblasts, which stimulates osteoclast activation and accelerates osteolytic destruction, perpetuating a vicious cycle (198). In addition, this pathway induces activation of cancer-associated fibroblasts (CAFs) leading to ECM remodeling and thus formation of pro-metastatic TME, but this also provides an approach for anti-fibrotic therapy (199). The pathway also regulates the self-renewal capacity of CSCs, causing them to be resistant to chemotherapy and thus promoting metastasis (200). Tumor-associated macrophages (TAMs) predominantly exhibit an M2-like phenotype in TME, which has been shown to promote tumor metastasis and progression (201). In contrast, the ligand SHH in the Hh signaling pathway induces TAM M2 polarization, resulting in a decrease in the expression of CXCL9 and CXCL10 leading to a significant down-regulation of CD8+ T cells infiltrating into the TME, which ultimately leads to an attenuation of the immunosuppressive function (61). Interleukin-4 (IL-4) is crucial in inhibiting anti-tumor immune responses and facilitating tumor cell proliferation (202), while the Hh pathway enhances IL-4 expression by stimulating T-helper 2 (Th2) cells transcription (203). Inhibition of the Hh pathway was observed to decrease PD-L1 expression and increase CD8+ lymphocyte expression, thereby enhancing anti-tumor activity (204). For targeted therapy against the Hh pathway, SMO and GLI inhibitors are currently important therapeutic options and have achieved good efficacy in the clinical treatment of LC (205, 206).

### AMPK pathway

AMPK, a serine/threonine kinase composed of regulatory β and γ subunits and a catalytic α subunit, acts as an energy sensor sensitive to the AMP/ATP ratio (62–64). It regulates energy homeostasis and metabolic stress responses, influencing cell proliferation, growth, stress response, autophagy and cell polarity to inhibit tumorigenesis (62–64).

AMPK acts mainly through oxidative phosphorylation and regulation of malignant tumor metabolism (63). In particular, AMPK activates p53 thereby delaying the cell cycle and can induce apoptosis (207). Resveratrol (RSV) enhances nerve growth factor receptor (NGFR) expression by modulating mRNA levels and the stability of mRNAs and proteins, thereby promoting AMPK phosphorylation and inhibiting mTOR phosphorylation, offering a potential targeted therapy for NSCLC (208). Metformin can activate AMPK to inhibit mTOR phosphorylation and suppress cell proliferation, but its antiproliferative effects appear to be independent of Liver kinase B1 (LKB1) (30). Metformin-activated AMPK at the S655 site phosphorylates downstream PHF2 to promote epigenetic H3K9me2 demethylation during EMT, thereby inhibiting LC metastasis (209). Metformin-activated AMPK recruits anti-inflammatory factors, including IL-1β, IL-6, TNF-α and NF-kB, resulting in reduced vascular endothelial growth factor levels (210, 211). LKB1 mutation is prevalent in NSCLC and crucial for activating AMPK family kinases (212). SIK1 and SIK3 are two tumor suppressor kinases of the AMPK family, and it has been found that in Kras-driven LC, the results of LKB1 deletion and SIK1/SIK3 deletion in regulating gene expression are highly overlapping and LKB1 deletion seems to activate the IL6/JAK/STAT pathway, which gives us a new direction for the treatment of LKB1 mutant LC (213). In addition, phosphorylation of AMPK promotes phosphorylation of downstream acetyl-coenzyme A carboxylase (ACC), thereby inhibiting lipid synthesis, a crucial process for nutrient acquisition in cancer cell growth and proliferation (63). The Warburg effect is profound in cancer metabolism by affecting glucose, amino acid, and lipid metabolism (214). Previous studies have found that β-elemene can activate AMPK pathways to counteract the Warburg effect in LC (215). This finding indicates that various signaling pathways may activate the AMPK pathway, either directly or indirectly, to influence the Warburg effect. The findings indicate that the AMPK pathway significantly impacts LC development.

The AMPK pathway influences LC metastasis by modulating energy metabolism, the immune microenvironment, and various other factors (63, 216). This pathway reduces VEGF expression by inhibiting HIF-1α, which inhibits tumor angiogenesis (216). Sustained activation of AMPK can inhibit protein synthesis by inhibiting the mTORC1 pathway or phosphorylating eEF2k, which can indirectly inhibit tumor cell proliferation (217, 218). TAMs is important for cancer cell proliferation, invasion, metastasis and angiogenesis (219). Studies have shown that astragaloside (AS-IV) can inhibit M2 polarization of TAM by targeting the AMPK signaling pathway, thereby inhibiting the progression and metastasis of LC (220). Most activated immune cells derive part of their energy from glycolysis, such as macrophages, neutrophils, B cells, dendritic cells, etc, and AMPK can promote cellular catabolism to inhibit immune cell activation (65). The LKB1-AMPK pathway is crucial for T cell differentiation and function through its regulation of metabolic reprogramming (221). In patients with KRAS and LKB1 co-mutant lung cancer, autophagy in cancer cells increases acetyl-coenzyme A (acetyl-CoA) levels, inducing EMT and promoting metastasis through the acetylation of the transcription factor Snail. CAMKK2 and ACLY inhibitors have been shown to effectively reduce cancer cell metastasis by targeting the autophagy/acetyl-CoA axis (222). Tregs contribute to cancer immunosuppression by expressing various immunomodulatory cytokines and inhibitory receptors. Conversely, the AMPK pathway can suppress PD-1 expression in Tregs via the HMGCR/P38 MAPK/GSK3β axis, thereby boosting anti-tumor immunity and offering a potential combination therapy for LC treatment (223).

## Importance of the interconnectedness of these pathways

**Figure 1:**
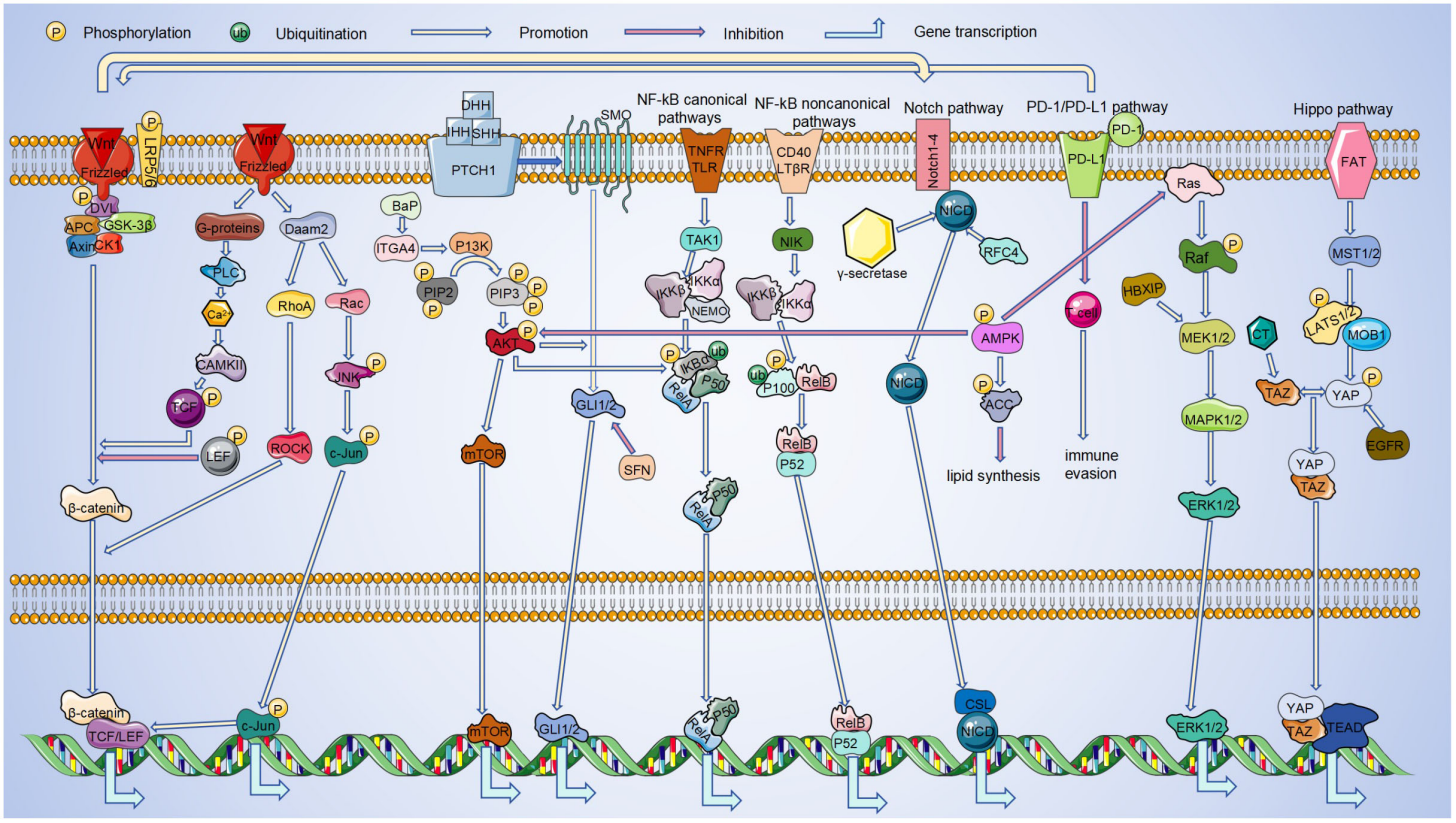
Linkages between pathways in LC. During the occurrence and development of LC, it is regulated by signaling pathways such as Wnt, PI3K/Akt, Notch, PD-1/PD-L1, NF-κB, Hippo, MAPK, Hedgehog, AMPK. And these pathways can be linked to each other. Among these signaling pathways, Hippo and AMPK pathways inhibit LC progression. The remaining pathways play an oncogenic role in LC progression. By interacting in the cytoplasm and nucleus, these pathways can ultimately regulate the transcription of target genes.

The linear progression of the Wnt, PI3K/Akt, Notch, PD-1/PD-L1, NF-KB, Hippo, MAPK, Hedgehog and AMPK pathways has been summarized in **Figure 1**. However, there has been a great deal of research on the linkage of these pathways showing that these signaling pathways are intimately connected by directly or indirectly interfering with each other (224, 225).

Wnt signaling has been shown to influence YAP expression and modulate Gli3 via the Hh pathway (27, 73). In addition, it can interact with Notch (118), and PD-L1 (76) signaling. Most RAS proteins predominantly exist in a GTP-bound state primarily due to mutations that enhance their stability, conferring structural activity and resistance to exogenous growth factors like EGFR. Thus, such K-RAS proteins appear to cause tumor cells to be less sensitive to ErbB-targeting drugs, including cetuximab or panitumumab (226, 227). The PI3K/AKT pathway activation enhances the Hh pathway’s involvement in LC progression (98). Wnt/β-catenin enhances Notch signaling, which reciprocally influences the Wnt/β-catenin pathway through Nrarp regulation (118). Similarly, Wnt can be activated not only by MAPK (79), but also regulated by PD-L1 (76). Activation of AMPK inhibits activation of PI3K/Akt (228) and Ras (229), in addition to it also promotes the biological function of NF-KB (210, 211). PI3K/AKT plays a synergistic role with MAPK in the self-renewal of lung CSCs (55). Hippo not only enhances MAPK function (133), but also inactivates Ras (170). PD1/PD-L1 activation involves the NF-KB pathway (132), and is regulated by the Hippo pathway (133).

The interplay of these pathways is crucial in cancer formation and progression. Although feedback loops are a fundamental part of carcinogenesis, their impact is indeed significant, suggesting that fine-tuning of one link may also affect the whole.

## Conclusion

This article examines the pathophysiological roles and interactions of various signaling pathways in LC. Pathophysiological studies have demonstrated that dysregulated signaling pathways significantly contribute to LC by enhancing cell proliferation and metastasis, while their interactions and feedback loops may suppress cell differentiation and apoptosis (224, 225).

In past studies, pathways that play a role in tumors continue to be unearthed, bringing our understanding of targeted therapy as a tool to new heights. Studies have shown that these pathways act in connection with each other rather than in isolation, with changes in one pathway acting as a chain reaction, leading to changes in another pathway (230). The growing research on signaling pathways highlights the critical need to understand their interactions, offering diverse strategies for cancer treatment. Dysregulation of several pathways is involved in the process of LC occurrence and development, mainly including Wnt (22), P13/Akt (23), Notch (25), PD-1/PD-L1 (4), NF-KB (26), Hippo (27), MAPK (17), Hedgehog (29) and AMPK (30).

A thorough investigation into the etiology, causative factors, and clinical treatments for LC is essential to develop a comprehensive and effective treatment strategy (4). However, if we only target a single gene in the treatment of LC, we often fail to achieve the expected efficacy, in which the complex etiology of LC has a significant impact. Furthermore, “cocktail therapy,” which combines multiple drugs, has proven more effective in treating the disease. In conclusion, exploring epigenetic mechanisms in LC development and progression offers unexpected insights, potentially enhancing therapeutic options and improving early diagnosis and treatment (37, 231).
